# A new method of identifying target groups for pronatalist policy applied to Australia

**DOI:** 10.1371/journal.pone.0192007

**Published:** 2018-02-09

**Authors:** Mengni Chen, Chris J. Lloyd, Paul S. F. Yip

**Affiliations:** 1 Wittgenstein Centre for Demography and Global Human Capital, Vienna University of Economics and Business, Vienna, Austria; 2 Department of Social Work and Social Administration, The University of Hong Kong, Hong Kong SAR, China; 3 Melbourne Business School, University of Melbourne, Melbourne, Australia; TNO, NETHERLANDS

## Abstract

A country’s total fertility rate (TFR) depends on many factors. Attributing changes in TFR to changes of policy is difficult, as they could easily be correlated with changes in the unmeasured drivers of TFR. A case in point is Australia where both pronatalist effort and TFR increased in lock step from 2001 to 2008 and then decreased. The global financial crisis or other unobserved confounders might explain both the reducing TFR and pronatalist incentives after 2008. Therefore, it is difficult to estimate causal effects of policy using econometric techniques. The aim of this study is to instead look at the structure of the population to identify which subgroups most influence TFR. Specifically, we build a stochastic model relating TFR to the fertility rates of various subgroups and calculate elasticity of TFR with respect to each rate. For each subgroup, the ratio of its elasticity to its group size is used to evaluate the subgroup’s potential cost effectiveness as a pronatalist target. In addition, we measure the historical stability of group fertility rates, which measures propensity to change. Groups with a high effectiveness ratio and also high propensity to change are natural policy targets. We applied this new method to Australian data on fertility rates broken down by parity, age and marital status. The results show that targeting parity 3+ is more cost-effective than lower parities. This study contributes to the literature on pronatalist policies by investigating the targeting of policies, and generates important implications for formulating cost-effective policies.

## Introduction

After a prolonged post-war fertility decline, many developed countries saw a recovery of fertility in the 1990s to early 2000s. Some of these countries introduced pronatalist incentives over the same period. Were these effective? Perhaps fertility would have recovered anyway even without policy intervention. Various alternative explanations of fertility recovery have been identified in the literature, including: tempo distortion [1,2]; delayed birth recuperation [3]; economic prosperity prior to the 2008 global financial crisis [2]; a possible reversal of the relationship between socioeconomic development and fertility trends in highly developed countries [4].

The key problem in estimating the effect of pronatalist policy on fertility is that other factors can drive them both, giving the illusion of causation. Economists would say that the policy change is *endogenous*. As just one example, in 2010 Japan introduced the universal child benefit but abolished it two years later, due to the financial effects of the 2011 tsunami [5,6]. The reduced fertility that followed may have been due to psychological effects of the tsunami, not the reduction in pronatalist incentive. More generally, the most obvious confounding factor in assessing pronatalist policy is the general attitude of a country towards fertility [7]. A more positive general attitude will tend to drive both fertility and government policies with that aim. It would seem nigh on impossible to control for this effect.

### Empirical studies on pronatalist policy effects

In Australia, a strong pronatalist narrative began around 1995, presaging the introduction of the 1996 maternity allowance and increasing benefits over the ensuing 12 years. Existing studies suggest that the policies had a small positive impact on fertility in Australia, while being very costly [8–13]. These results are consistent with international findings [8,14–16]. Conscious of the modest effect and high expense, many governments are more reluctant to take pronatalist action [17], especially after the 2008 financial crisis. This raises the question of whether a more targeted policy might influence the fertility rate more cost effectively.

Frejka [18] examined pronatalist measures in Czechoslovakia concluding that its success was due to accurate targeting of the policy, indicating that a well-tailored policy might make a big difference. In response to the 2008 global financial crisis, the original universal child benefits in the UK became means-tested [5,19]. Indeed, many pronatalist governments started to replace universal programs with targeted ones [20–22], indicating a growing need for a more elaborate policy design.

Notwithstanding the difficulties of endogeneity, empirical studies suggest that policies had heterogeneous impacts on different population subgroups, especially groups defined by parity. In Hungary, child-related benefits had a positive effect on second and third births [23]. Child allowances in the UK were associated with early motherhood as well as third and fourth births [24]. In Quebec, Milligan [25] found that cash benefits had a strong impact on third or higher order births. Studies evaluating the universal Baby Bonus in Australia found that first births seemed less affected than second or third births [9–11]. Gauthier and Hatzius [26] evaluated cash benefits in 22 developed western countries and found that policies targeting first births had a larger effect on the TFR than targeting third births. But they pointed out that, because the first birth group is so large, targeting them would be costly and may not result in a “greater bang for the government’s buck.” In terms of identifying a target group, the existing literature seems to imply, but not address, a hidden trade-off between the cost and the impact of policies.

How can we identify groups whose targeting will influence TFR in a cost effective way i.e. groups that give the largest “bang per buck” [27]? In the current context, “buck” is the cost of targeting that group which will be proportional to the size of that group, and “bang” is the change in TFR obtained from a likely change in group fertility rates. Likely changes in group fertility rates can be roughly estimated by historical variations and does not necessarily require any econometric modelling. And the impact of group fertility rate on TFR depends on current population structure.

The plan of the paper is as follows. We begin by giving a brief history of pronatalist policies in Australia, since Australian data is used to illustrate the method. This data is broken down by mother’s age, marital status and parity, which has not been available previously. To estimate the impact of group fertility rate on TFR, we construct a simple stochastic model for TFR and calculate the elasticity with respect to each group fertility rate. We measure the likely changes in fertility rates of each group from their historical trends. Based on all this information, we calculate the benefit-cost ratio for targeting each group. A comparison of the benefit-cost ratio is made to provide a benchmark for policy makers to evaluate the cost-effectiveness of pronatalist programs.

### Australian fertility trends and policies from 1990–2014

**Fig 1:**
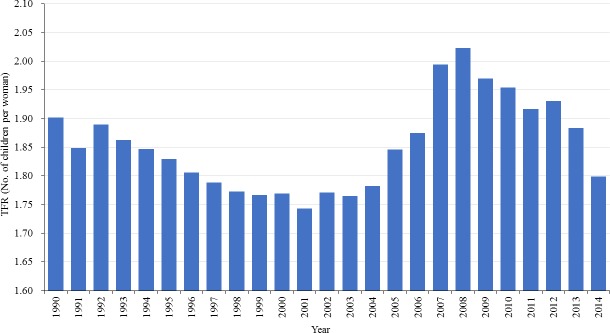
The TFR of Australia from 1990–2014.

Fig 1 displays the historical pattern of TFR for Australia from 1990 to 2014 with major changes to pronatalist policy indicated. Fertility rates declined strongly from 3.55 in 1960 to replacement of 2.06 in 1976 and have remained below replacement since then, the lowest recorded level being 1.73 in 2001.

Faced with declining fertility in the 1990s, the Australian Government reintroduced the maternity allowance, a means-tested lump-sum payment of AUD$840 per child. The Child Care Benefit was introduced in 2000, whereby families that passed a means test could receive an hour rate up to 50 hours per week. In 2002, the First Child Tax Refund was available to parents following the birth of the first child and the payment amount depended on the income of the primary carer [28]. Set against a backdrop of pronatalist rhetoric, these two payments were replaced by the much more generous universal Baby Bonus in 2004 of AUD$3,000 per baby. At the same time, the non-means tested Child Care Tax Rebate was put forward, worth up to $4000 per annum and changed to a lump sum payment in 2006. The Baby bonus was raised twice to AUD$4,000 in 2006 and to AUD$5,000 in 2008. In 2008, the Child Care Tax rebate increased to 50% of child care costs (worth up to AUD$7,500) with inflation indexation.

The untargeted nature of the Baby Bonus invited criticism [9,13,29] and the financial crisis intervened. Thereafter, pronatalist policies have largely been unwound. In 2009 inflation indexation of the Baby Bonus was frozen and means testing introduced, probably in response to financial pressure [30]. From 2011, indexation of the Child Care Rebate was also frozen. In July 2013, the Baby bonus was reduced and targeted more at first births. Finally, on 1 March 2014, it was abolished and replaced by less generous schemes.

Fig 1 seems to suggest that the policy was effective, as the TFR rebounded with the enhancement of pronatalist policies. However, there are other reasons why fertility could increase up to 2008 and then decrease. The same pattern has been observed in many other developed countries, not all of which explicitly adopted pronatalist policies. Some scholars have attribute this to the diminishing tempo effect and the changing parity composition of the female population [3,8]. Kippen [31] investigated Australia’s fertility decline in the 1990s and attributed most of it to the delay in first and second births; higher-order birth rates remained relatively constant over the decade. Parr and Guest [8] studied Australia’s fertility increase in the early 2000s, and argued that it was more likely a result of the changing age-parity distribution of women at childbearing age, than of the success of the pronatalist measures. More specifically, they argued that it was the increasing proportion of women in the later childbearing age and at low parities who produced the rise in fertility, as these groups of women have higher childbearing propensities. These two studies highlight the role of changing childbearing patterns and distribution of women in the determination of Australia’s fertility, which have important implications for predicting and managing the future trajectory of the TFR.

Budgetary limitations, especially after the recent financial crisis, call for a targeted policy which can cost-effectively influence the fertility rate. How do targeted policies fit with the changing childbearing patterns and age-parity composition of the female population in Australia? Unlike previous research, most of which directly examined the apparent impact of pronatalist actions, this study will try to explore the potential targeting of policies.

## Methods

### Data

Motivated by the cited research of Kippen [31] and Parr and Guest [8], we will define subgroups by mother’s age and number of prior children (called parity). In addition, we are also able to break the data down by marital status.

To estimate group-specific fertility rates, we required the number of women in the population and the number of babies born, broken down by age, marital status and parity. Such data were not publically available, though recorded. The female population data were obtained from National Information Consultancy Services of the Australia Bureau of Statistics (ABS) for the census years 1996, 2006 and 2011. Based on censuses, numbers of female residents in Australia are cross-classified by marital status, 5-year age-groups and the number of children ever born (from 0 up to 6+). As parity information was not recorded in the 2001 census, the year 2001 is not included in the analysis.

The national birth data was provided by the demography section of New South Wales section of ABS Information Consultancy Services. All recorded births in the years 1997, 2007, 2012 (i.e. one year later than the census data) are classified by marital status, 5-year age-groups and birth order (1st, 2nd, 3rd or 4th+ children). Instead of using the number of mothers giving birth, the number of babies is used in each cross-category (the difference between the two being attributed to multiple births), as this reflects actual rather than intended fertility rates. Though the data are of generally high quality, some data cleaning and adjustments were necessary and details are in S1 Appendix. Both the female population data and the birth data can be found in S1 File.

The stochastic model we develop also requires age-specific marriage and divorce rates. The marriage rates are calculated by dividing registered marriages (including remarriages) by the number of unmarried females, broken down by 5-year age group. Age-specific marriage rates for 1997 were extracted directly from the Marriages and Divorces 1998 report. The numbers of marriages for 2007 and 2012 were provided by ABS. The age-specific divorce rates (the number of divorces per 1000 married women) for years 1996, 2006, and 2011, were extracted from ABS report 3310.0 Marriages and Divorces, Australia, 2012.

### The parity transition model

A stochastic model is constructed for TFR, which tracks transitions in marital status and parity of women over her reproductive life. Unlike many Asian countries, marriage in Australia is not a social prerequisite to childbearing, and out-of-wedlock births currently account for 35% of all births [32] but less than 5% in Asian countries, such as Taiwan, Japan and South Korea. Nevertheless, fertility patterns are different between married and unmarried women, and so marital status of women is tracked in the model. Since the birth data only recorded the parity up to 4+, parity 4+ is treated as an absorbing state. This would have very little influence on our results, as the proportion of mothers with more than 4 children in Australia is very small.

A Markov chain is used to model the transition dynamics. Marital status has two states, namely unmarried (including single, cohabiting, and divorced) and married; parity has five states, namely 0 to 4+. Several simplifying assumptions are made: one woman can give birth to one child at most each year, but an appropriate adjustment is made to take account of multiple births; marriage (or divorce) and childbirth do not happen in the same year; the fertility rates of the remarried and first-married women are the same.

**Fig 2:**
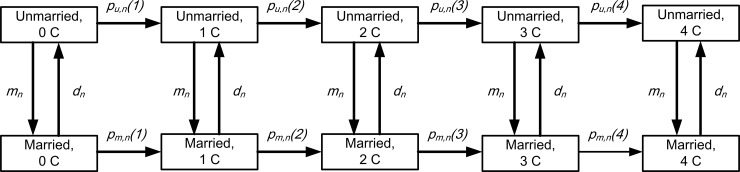
The Markov transition model.

Fig 2 describes the model. Vertical arrows represent marriage and divorce, with *m_n_* denoting the probability of an unmarried woman marrying at age *n* and *d_n_* the probability of a married woman divorcing at age *n*. Horizontal arrows represent parity transitions. For married women, the probability of having a *j*th child at age *n* in a given year is denoted by *p*_*m*,*n*_*(j) j* = 1,…,4 and for unmarried women by *p*_*u*,*n*_*(j)*. Together with the probabilities of marriage and divorce, there are 2+4+4 = 10 parameters governing transitions at any age. As women’s age is categorized into seven 5-year age groups (covering age 15–49), there are 10×7 = 70 parameters in all.

Based on this model, it is possible to write down an expression for the TFR in terms of these parameters. The formula is expressed in matrix notation (for details see S2 Appendix). Letting *θ*_1_,*…*,*θ*_70_ denote the 70 marriage, divorce and group fertility parameters in the transition model, TFR(*θ*_1_,*…*, *θ*_70_) is a function of these parameters.

### Fertility elasticity

Having a model for TFR allows us to measure the impact on TFR from small hypothetical changes in group-specific fertility rates. It should be noted that changes in a group-specific fertility rate may not reflect a change in fertility intention. In reality, women may change the timing of childbearing, without changing the number of children intended or desired. This important point will be revisited in the Conclusion and Discussion section. For now, since few intend to have more than two children, changes in the third-and-high-parity fertility rates are more likely to represent real changes in fertility intention.

Elasticity, a concept in economics, is a measure of the percentage change of quantity demanded per 1% change in the price. Analogously, the elasticity of TFR with respect to parameter *θ*_J_ is computed as

$$\eta_{J}=\frac{\partial TFR}{\partial \theta_{J}}\times \frac{\theta_{J}}{TFR}$$ (1)

and measures percentage change in TFR per 1% change in parameter *θ*_J_. Since TFR (*θ*_1_,…, *θ*_70_) is a very complex function, derivatives are computed in Matlab. The focus of the analysis is on the 56 fertility parameters rather than the marriage and divorce rates. It should be emphasized that elasticity is more meaningful than a raw partial derivative, as the former consider the proportional change of the parameter rather than an absolute change. For example, the fertility rate for parity 2 of the 45–49 year-old unmarried women is at the level of 0.001 children per woman, so it is more reasonable to assume 1% increase than an absolute increase of 0.1 child per woman for this group.

### Likely change in the group fertility rate and likely impact on TFR

The fertility elasticity *η*_J_ measures the change in TFR for a 1% change in the fertility rate of group J. But to compare different groups in their influence on TFR, we should try to estimate effect of a *likely change* in group fertility, rather than that induced by the same hypothetical change of 1% for each group.

This is the only part of the methodology that requires historical rather than current data. By looking at historical variations in group fertility rates, we might estimate plausible future changes in these rates. In this paper, we will use a rather crude measure, namely the historical variability of each group fertility rate, for the current application across four time points 1997, 2002, 2007 and 2012. And since elasticity is concerned with proportional changes, it makes sense to measure variability in proportional terms i.e. to use the *coefficient of variation (CV)*. The likely change in TFR from targeting group J is then estimated by multiplying the elasticity *η*_J_ by the coefficient of variation CV_J._ Therefore, the likely impact would be *η*_J_×CV_J_.

In using CVs of historical group fertility rates as a measure for their propensity to change, we are ignoring the direction of the historical variations. The implicit argument then is that if group fertility rates are increasing over time, policy might help facilitate this trend; if they are decreasing over time then policy might reverse that trend. This is indeed the typical purpose of pronatalist policy. Other more sophisticated measures for the propensity to change might be considered in future work.

### Benefit to cost ratio

While it is not feasible to put a dollar value on marginal increases in fertility, it is practically important to compare the effect of pronatalist policies to their cost. The monetary cost of a pronatalist program is hard to measure, and depends on the details of the program. However, as the vast majority of such programs deliver benefits *per child*, it would be reasonable to argue that the cost of a policy for a particular group is *proportional to* the number of babies born in that group, which we denote by *B*_J_.

Different subgroups can be compared in their cost-effectiveness, via the ratio *η*_J_×CV_J_/*B*_J_. Since here we do not put a dollar value on likely impact on TFR (measured by *η*_J_×CV_J_) and policy costs are only proportional to *B*_J_, these ratios are on an arbitrary scale. Largely for cosmetic reasons, we do not use the absolute number of babies born to a certain group (i.e. *B*_J_) but the proportion of babies in this group to the overall number of babies–denoted by *b*_J_ (= *B_J_*/∑*B_J_*).

## Results

### Observed changes in the pattern of group specific fertility rates

**Fig 3:**
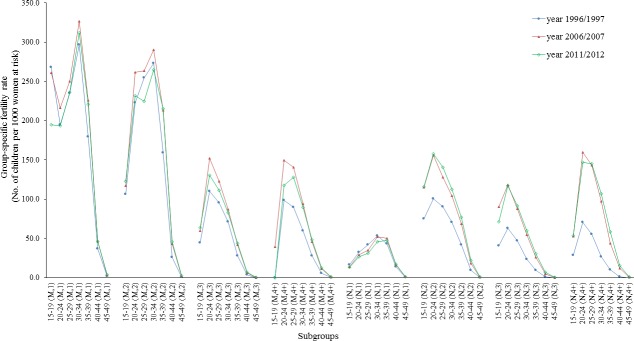
Age parity specific fertility rate by marital status. Notes: M refers to married women; and N refers to unmarried women.

Fertility rates for all subgroups by marital status, age-group and parity (56 in all) for the years 1996/1997, 2006/2007, and 2011/2012 were calculated and are displayed in Fig 3. Note that these fertility rates are conditional on women’s parity level. Each is expressed as the number of children per 1000 women of a certain group. The only other analysis of age-parity specific fertility rates in Australia is Kippen [31] who ignored marital status. Here we have further demonstrated that the differences between the married and the unmarried are most noticeable at parity 1. This suggests that marriage is more associated with the decision to have children. The differences are much less at parity 2 (i.e. conditional on having one child) and nearly disappear at higher parities. Fertility rates at parity 3+ are generally low, reflecting the two-child norm in Australia [31].

There have been changes over time. Nearly all rates increased from 1997 to 2007, a period where the economy was prospering and pronatalist policy was introduced and strengthened (Fig 1). And over 1997–2007 the largest increases were for parity 2+, and especially for unmarried mothers. This increase may be related to a social climate more open to non-marital childbearing in Australia; or on the other hand, it may also imply some impacts of the pronatalist action during those years [9,11]. However, this levelled off between 2007 and 2012. As argued earlier, attributing causation to any of these patterns is fraught with difficulty.

### Elasticity of TFR with respect to fertility parameters

Table 1 shows the TFR elasticities η_J,_ computed using Matlab, with respect to 70 parameters for the year 2012 only. Details of the elasticity calculations can be found in S2 Appendix. Since active policies to affect marriage or divorce rates are not on the table in Australia, the elasticities with respect to marriage and divorce rates at each age are only included for completeness.

**Table 1:** Elasticities of TFR with respect to 70 key parameters.

age group	marriage rate	divorce rate	marital fertility rate				non-marital fertility rate			
			parity 1	parity 2	parity 3	parity 4+	parity 1	parity 2	parity 3	parity 4+
**15–19**	0.007	-0.000	0.001	0.000	0.000	0.000	0.041	0.003	0.000	0.000
**20–24**	0.059	-0.002	0.011	0.004	0.001	0.000	0.061	0.016	0.006	0.000
**25–29**	0.113	-0.007	0.033	0.018	0.012	0.002	0.044	0.019	0.013	0.002
**30–34**	0.059	-0.007	0.049	0.040	0.037	0.007	0.037	0.017	0.012	0.004
**35–39**	0.010	-0.003	0.029	0.039	0.033	0.011	0.023	0.014	0.008	0.004
**40–44**	0.001	-0.000	0.005	0.009	0.006	0.004	0.006	0.005	0.002	0.001
**45–49**	0.000	-0.000	0.000	0.001	0.000	0.000	0.000	0.000	0.000	0.000

Here the focus is on the elasticities with respect to the 56 subgroup fertility rates. To visualize the pattern of elasticities, Table 1 uses color (highest = green and lowest = red). The overall patterns for 2007 and 1997 are extremely similar and not displayed. There are clear differences between the married and unmarried, especially at lower parities. For unmarried, the highest elasticities are for parity 1 across the entire 15–34 age groups. For the married, high elasticities occur mostly in those aged 30–39 and for transitions to parity 1, 2, and even 3. All of these elasticities are individually small. Significant impacts on TFR would only follow from changing several of these fertility rates by significant amounts.

### Likely change in the group fertility rate and likely impact on TFR

Table 2 shows the coefficient of variation CV_J_ of each group fertility rate over the four time periods using the same color scale as Table 1. Overall, fertility rates of the unmarried are apparently more changeable than of the married, especially at higher parities and ages. Most pertinently, it appears that, for married women, fertility rates in the main childbearing ages of 25–34 and at the most common parities of 1 and 2 have shown the least historical variations. This indirectly confirms previously mentioned research suggesting that third and fourth births are more responsive to monetary incentives [10,11,24,33,34].

**Table 2:** The coefficient of variation of 56 key parameters (in %).

Age group	Marital fertility rates				Non-marital fertility rates			
	Parity 1	Parity 2	Parity 3	Parity 4+	Parity 1	Parity 2	Parity 3	Parity 4+
**15–19**	14	6	15	128	10	19	31	27
**20–24**	5	7	13	17	8	20	27	32
**25–29**	3	7	10	18	13	19	28	39
**30–34**	4	4	9	19	7	20	37	50
**35–39**	10	14	20	23	6	25	45	61
**40–44**	11	24	28	30	10	35	62	70
**45–49**	34	51	53	53	30	32	107	81

In Table 3, we display η_J_×CV_J_ which measures the % change in TFR for a likely % change in the group fertility rate, rather than an arbitrary 1% change. The most influential groups are 35–39 year-old married women at parity 3 and parity 2. For unmarried mothers, the most influential segments are first births for mothers under 30, and second and third births for older mothers aged 25–39. If we imagine a completely untargeted pronatalist policy such as the universal Baby Bonus in Australia, the greener cells would tend to contribute more to any increase in TFR.

**Table 3:** Likely impacts on TFR from likely variations in the 56 key parameters (impact in %).

age group	marital fertility rates				non-marital fertility rates			
	parity 1	parity 2	parity 3	parity 4+	parity 1	parity 2	parity 3	parity 4+
**15–19**	0.014	0.001	0.000	0.000	0.412	0.061	0.008	0.000
**20–24**	0.057	0.027	0.015	0.001	0.485	0.318	0.154	0.010
**25–29**	0.098	0.129	0.123	0.027	0.574	0.357	0.371	0.078
**30–34**	0.198	0.158	0.329	0.137	0.262	0.332	0.460	0.184
**35–39**	0.292	0.552	0.666	0.245	0.136	0.359	0.355	0.227
**40–44**	0.055	0.222	0.163	0.113	0.057	0.165	0.136	0.101
**45–49**	0.016	0.029	0.013	0.016	0.013	0.007	0.016	0.010

There are some important differences between Tables 1 and 3. For instance, the likely impact of married parity-1 fertility in the main childbearing ages of 25–29 is much lower than the elasticity Table 1 suggests, due to the relatively smaller historical variations in these parameters. On the other hand, the likely impact of unmarried parity 3 fertility is higher than elasticity suggests.

### Benefit to cost ratio

Table 4 shows patterns in the ratio of η_J_×CV_J_ in Table 3 to the proportion of births b_J_ in each group. Those with relatively higher benefit-cost ratios are the oldest age-groups and those unmarried (aged over 30) at higher parities.

**Table 4:** Benefit-cost ratio indices for 56 mother groups.

age group	marital groups				non-marital groups			
	parity 1	parity 2	parity 3	parity 4+	parity 1	parity 2	parity 3	parity 4+
**15–19**	8.83	2.06	1.83		13.63	14.15	20.24	0.61
**20–24**	2.15	2.04	4.09	1.08	8.66	11.91	19.42	4.00
**25–29**	0.95	2.16	6.32	3.82	13.47	13.61	30.24	9.75
**30–34**	1.97	1.57	8.50	8.76	8.42	17.13	46.53	20.90
**35–39**	8.28	9.97	22.31	15.90	7.30	27.72	53.98	35.19
**40–44**	8.50	22.42	29.58	22.56	10.17	42.52	68.85	45.34
**45–49**	33.17	64.35	66.81	45.51	36.04	50.90	148.3	66.26

As policies usually target parity, rather than the age or marital status of women, we calculated the benefit cost ratio for groups by parity. To do this, for each parity we divided the accumulation of the likely impact in Table 3 over age-and-marital-status groups by the accumulation of the proportion of births. The results are in Table 5. Comparisons of these ratios can help identify which parity might be targeted to maximize cost effectiveness. The results indicate that the most efficient policy target is parity 3 –four times more effective than parity 1. These represent 13.6% of births. Looking back at Table 3, we can see that the achieved changes will be mainly in the 30–39 age-group. Targeting births of parity 4+ is the next most cost-effective. Targeting parity 2 would be around half as effective as targeting parity 4+.

**Table 5:** Benefit\costs ratios by parity (integrated over age and marital status).

	parity 1	parity 2	parity 3	parity 4+
**Likely Change**	2.667	2.718	2.809	1.148
**% Births**	45.76%	33.34%	13.64%	7.23%
**BC ratio**	5.83	8.15	20.59	15.87

## Conclusion and discussion

Estimating the causal effects of pronatalist policy on TFR is difficult because policy changes are often endogenous. For instance, a positive national stance towards population growth may easily lead to both pronatalist incentives and increases in TFR that would have occurred anyway. So, policy effects tend to be over-estimated. At the same time, there is concern about the cost effectiveness of policy, which has motivated many countries to shift towards more targeted incentives. This highlights the need for research on how the TFR can be influenced in a cost-effective way. To fill this gap, this paper has made several contributions.

We suggest a new method for identifying population subgroups that are better targets of pronatalist policy. It involves measuring three factors: (1) the sensitivity of TFR to changes in group fertility rates, (2) the propensity of each group fertility to change and (3) the total cost of the policy for that group. Our final measure is (1)×(2)/(3). Only mother and birth count data by group and minimal time series data is required. Modelling of causality is side-stepped by focusing on the structure of the population. In our illustration, groups are defined by age, marital status and parity of the mother and we used data at three time points. This level of data has not been previously available in Australia.

To measure the sensitivity of TFR to changes in group fertility rates, we built a parity transition model that relates TFR to group fertility rates (as well as marriage and divorce rates) and calculated fertility elasticity which is originally an economic concept. It measures the percentage change in the TFR given 1% change in a certain group’s fertility rate and automatically takes account of the size and fertility rate of the group. However, it might reasonably be argued that changes in parity specific fertility rates could affect the timing of childbearing rather than desired family size. This is true in principle and if a particular parity fertility rate increased then later parity rates might decrease in response. The magnitude of this effect will surely depend on the public perceptions of whether the policy is going to persist over their planning horizon. So in order to realise the predictions of our elasticity model, long-term pronatalist policies should be adopted. Moreover, in reality only long-term policy, which is seen to be long term, will likely be effective anyway.

Besides, it should also be emphasized that the change in timing risk will surely be less for higher parities, that is, an increase in say parity 4+ fertility is much more likely to represent an increase in fertility intention than a change in parity 1 fertility. In other words, the diluting effect of potential changes in timing changes will decrease with parity. Consequently, any bias in our method due to timing is towards lower parities. So, if our method identifies higher parities as better targets (as previous empirical studied have) then the real effect may in fact be larger. This is precisely what we found in our analysis of Australian data–support for targeting parities 3 and 4+. Finally, timing effects will surely be less for older mothers and most of the fertility gains for parities 3 and 4+ are from older mothers.

To measure the propensity of each group fertility to change, we looked at historical variation of group fertilities. One quite crude method is to use coefficient of variation, the idea being that historical fluctuations, either increase or decrease, or mixed, are predictive of the magnitude of changes that are likely to occur in the fertility rate of that group. More sophisticate methods could be used for this step in the future. To measure the total cost of the policy, we assumed that the cost for targeting each group is proportional to the proportion of births in that group. If there were economies of scale, then some power of *b*_J_ could be used.

Applying the method to Australian data, the results show that the TFR is most mathematically sensitive to changes in the following fertility rates: parity-1 for the 15–34 year-old unmarried women; parity 1, 2, and 3 among the 30–39 year-old married women (see Table 1). But after taking account of propensity to change the pattern changes (Table 3). In Australia, the universal Baby Bonus has been found to incentivize third and higher parities in Western Australia and New South Wales [10,11]. Thus, to increase or maintain the TFR at a desirable level calls for a combination of different types of pronatalist policies, to remove obstacles faced by different subgroups.

There are some limitations in this study. Several minor assumptions are made to facilitate the computations, which consequently do not fully model the exact parity transition in real life. But the analysis can still shed light on Australia’s fertility trends, especially the roles of different subgroups in the group fertility rate (Table 2). Among unmarried women, the likely impacts on TFR are more spread across parities 1–3; whereas among the married, the likely impacts are more concentrated on the 30–39 year-old women who were at parity 2 and 3 (Table 3). Taking account of the policy cost, the benefit cost ratios look very different and the results suggest focusing on older age-groups, and especially higher parities for unmarried mothers (Table 4).

Since it is unrealistic in Australia to directly target people by age-group or marital status, we calculated the benefit cost ratios for groups only by parity. We found that targeting parity 3 is the most cost-effective and four times more cost-effective than targeting parity 1 (Table 5). This result agrees with empirical research across a wide range of nations. Existing studies show that higher-order births are more responsive to monetary incentives, possibly due to the economies of scale in a family: the marginal cost of having an additional child declines as the number of children increases. The analysis also shows that non-marital fertility rate plays an important role in the determination of the TFR. However, due to data limitations, non-marital fertility rates could not be differentiated between cohabiting and non-cohabiting unmarried women. Another important limitation is that other economic and socio-demographic factors such as employment status and educational attainment, are not used in the analysis. At present, it is not possible to obtain such data but it is intended to do so after the next Australian census.

## Supporting information

S1 Appendix (DOCX)

S2 Appendix (DOCX)

S1 File (XLSX)
