# Supplementary material for: A new method of identifying target groups for pronatalist policy applied to Australia
Source: PLoS One. 2018 Feb 9;13(2):e0192007. doi: 10.1371/journal.pone.0192007 (PMC5806865; doi:10.1371/journal.pone.0192007)
Supplement: S1 Appendix — (DOCX) [file pone.0192007.s001.docx]

**Data Cleaning**

**Adjustment for female population and birth data**

For the female population data from censuses 1996, 2006, 2011, age was recorded in every case and every year. However, roughly 5 to 6% (of women aged 15-49) in the three censuses did not record numbers of children ever born. Under the assumption that the missing parity was unrelated to age of the mother, records with missing parity were distributed across age groups proportional to the counts of parity-recorded females.

For the birth data in 1997, only 0.05% of the records (i.e. 119 out of 251,849) did not have the mother’s age stated. These were distributed in proportion to observed counts. All 251,849 records had parity of the child stated, as this was compulsory information on birth certificates in 1997. For the birth data in 2007, among 292,125 babies, 326 cases had the mother’s age unstated and 4,690 had the parity unstated. Records with missing age were distributed across age groups in proportion to observed counts and then records with missing parity were distributed across stated parities again in proportion to observed counts. This method of adjustment was also applied to the 2012 birth data, when 249 and 1,667 out of the total 309,578 births were age-unstated and parity-unstated respectively.

A further adjustment was applied to make the parity distribution of multiple births consistent over the three time points. That is, multiple births born to a mother who had *j* children were all assigned to the parity *j+1*. Due to the limited parity information for the birth data, the parity specific fertility rates were calculated up to parity 4+.

Because there were very few births of fifth and high order, the analysis only modelled the transition up to parity 4+ when calculating $p_{u,n}\left( 4 \right) and p_{m,n}\left( 4 \right)$. This means that births of parity 4 and higher were used as the numerator while the number of married/unmarried women with 3 children as the denominator to compute the fertility rate of parity 4+ by marital status.

**Adjustment for age-parity specific fertility rates**

The age-specific fertility rates (ASFR) were calculated based on 5-year categories, using the birth data in years 1997, 2007, and 2012 as the numerator and the female census data in 1996, 2006, and 2011 as the denominator. S1 Table 1 shows the discrepancies between the calculated results (the ASFR multiplied by 5) and the 5-year accumulated results of the official single-year ASFR. In order to reproduce TFR more closely to the official figure, the ratios of the self-calculated column to the official column in Table 5 were applied to adjust age-parity specific fertility rates.

**S1 Table 1 The discrepancy between self-calculated and official ASFR**

| **Age of Mother** | **1997** | |  | **2007** | |  | **2012** | |
| --- | --- | --- | --- | --- | --- | --- | --- | --- |
|  | **Self-calculated** | **Official** |  | **Self-calculated** | **Official** |  | **Self-calculated** | **Official** |
| **15-19** | 101 | 99.9 |  | 89.8 | 85.5 |  | 83.7 | 79.4 |
| **20-24** | 320.9 | 313.7 |  | 320.7 | 293.1 |  | 294.9 | 265.4 |
| **25-29** | 605.1 | 574.2 |  | 607.9 | 551.5 |  | 567.2 | 513 |
| **30-34** | 542.1 | 537.7 |  | 663.5 | 650.2 |  | 684.4 | 633 |
| **35-39** | 232.5 | 223.8 |  | 362.9 | 344.6 |  | 361.3 | 360.2 |
| **40-44** | 39 | 37.4 |  | 65.6 | 66.1 |  | 79.6 | 74.4 |
| **45-49** | 1.3 | 1.4 |  | 3.3 | 3.3 |  | 4.5 | 4.9 |
| **TFR** | 1842 | 1788 |  | 2114 | 1994 |  | 2076 | 1930 |

Sources: the official ASFRs are from ABS
