# Supplementary material for: A new method of identifying target groups for pronatalist policy applied to Australia
Source: PLoS One. 2018 Feb 9;13(2):e0192007. doi: 10.1371/journal.pone.0192007 (PMC5806865; doi:10.1371/journal.pone.0192007)
Supplement: S2 Appendix — (DOCX) [file pone.0192007.s002.docx]

**Functional relationship of TFR to marriage, divorce, and fertility rates**

According to the transition model, a woman at age *n* might be in one of 10 states, i.e. two levels of marital status and 5 level of parity. Let $u_{J}$(respectively $m_{J}$) be a binary indicator of being unmarried/married with J children. The binary states were listed in the order $(u_{0},m_{0},u_{1},m_{1},u_{2},m_{2},u_{3},m_{3},u_{4+},m_{4+})$. The probability vector of the 10 binary states at age *n* was denoted as below:

$\pi_{n}$=($\pi_{u,n}\left( 0 \right)$,$\pi_{m,n}\left( 0 \right)$,$\pi_{u,n}\left( 1 \right)$,$\pi_{m,n}\left( 1 \right)$,$\pi_{u,n}\left( 2 \right)$,$\pi_{m,n}\left( 2 \right)$,$\pi_{u,n}\left( 3 \right)$,$\pi_{m,n}\left( 3 \right)$,$\pi_{u,n}\left( 4 \right)$,$\pi_{m,n}\left( 4 \right)$).

Initially all women begin in state *u*_0_ and so $\pi_{15}=(1,0,0,0,0,0,0,0,0,0)$, meaning that all women are unmarried with 0 children at age 15.

For each age *n*, $p_{m,n}(j$*)* with *j = 1, 2, 3, 4* represents the transition probability of the married from parity *j-1* to parity *j*;$p_{u,n}(j$*)* with *j = 1, 2, 3, 4* represents the transition probability of the unmarried from parity *j-1* to parity *j*. The probability of a woman marring at age *n* is $m_{n}$ and of getting divorced at age *n* is $d_{n}$. This model (Fig 2) described how women move potentially through the 10 states and was summarized by the matrix transition equation:

$$\pi_{n+1}=\left( \begin{matrix} \pi_{u,n+1}(0) \\ \pi_{m,n+1}(0) \\ \pi_{u,n+1}(1) \\ \pi_{m,n+1}(1) \\ \pi_{u,n+1}(2) \\ \pi_{m,n+1}(2) \\ \pi_{u,n+1}(3) \\ \pi_{m,n+1}(3) \\ \pi_{u,n+1}(4) \\ \pi_{m,n+1}(4) \end{matrix} \right)=T_{n}\left( \begin{matrix} \pi_{u,n}(0) \\ \pi_{m,n}(0) \\ \pi_{u,n}(1) \\ \pi_{m,n}(1) \\ \pi_{u,n}(2) \\ \pi_{m,n}(2) \\ \pi_{u,n}(3) \\ \pi_{m,n}(3) \\ \pi_{u,n}(4) \\ \pi_{m,n}(4) \end{matrix} \right)=T_{n}\pi_{n}$$

where the transition matrix $T_{n}$ is given by:

$$\left( \begin{matrix} 1-p_{u,n}(1)-m_{n} & d_{n} & 0 & 0 & 0 & 0 & 0 & 0 & 0 & 0 \\ m_{n} & 1-p_{m,n}\left( 1 \right)-d_{n} & 0 & 0 & 0 & 0 & 0 & 0 & 0 & 0 \\ p_{u,n}(1) & 0 & 1-p_{u,n}(2)-m_{n} & d_{n} & 0 & 0 & 0 & 0 & 0 & 0 \\ 0 & p_{m,n}(1) & m_{n} & 1-p_{m,n}(2)-d_{n} & 0 & 0 & 0 & 0 & 0 & 0 \\ 0 & 0 & p_{u,n}(2) & 0 & 1-p_{u,n}(3)-m_{n} & d_{n} & 0 & 0 & 0 & 0 \\ 0 & 0 & 0 & p_{m,n}(2) & m_{n} & 1-p_{m,n}(3)-d_{n} & 0 & 0 & 0 & 0 \\ 0 & 0 & 0 & 0 & p_{u,n}(3) & 0 & 1-p_{u,n}(4)-m_{n} & d_{n} & 0 & 0 \\ 0 & 0 & 0 & 0 & 0 & p_{m,n}(3) & m_{n} & 1-p_{m,n}(4)-d_{n} & 0 & 0 \\ 0 & 0 & 0 & 0 & 0 & 0 & p_{u,n}(4) & 0 & 1-m_{n} & d_{n} \\ 0 & 0 & 0 & 0 & 0 & 0 & 0 & p_{m,n}(4) & m_{n} & 1-d_{n} \end{matrix} \right)..$$

Assuming that fertility for women aged 50 and over negligible, TFR could be computed from stationary probabilities at age 50 by

$TFR={(0,0,1,1,2,2,3,3,4,4)}^{T}\times\pi_{50}={(0,0,1,1,2,2,3,3,4,4)}^{T}\times T_{49}T_{48}\cdots T_{15}\pi_{15}$

This basic equation defined the relationship between TFR and the 70 parameters mentioned above and in the text. Due to lack of data on a finer scale, age was modelled through seven 5-year age-groups and the age-specific rates $m_{n}$*,*$d_{n}$*,* $p_{u,n}\left( j \right)$and $p_{m,n}\left( j \right)$ were assumed constant within each age-group.

For the 2011/2012 data, the state distribution of women at age 50 (i.e. at the end of their reproductive life) was calculated and is displayed in S2 Table 1. The TFR for 2012 calculated from these probabilities was 1.896.

**S2 Table 1 The state distribution of women at age 50**

|  | **0 child** | **1 child** | **2 children** | **3 children** | **4+ children** | **Total** |
| --- | --- | --- | --- | --- | --- | --- |
| **Unmarried** | 10.3% | 8.1% | 13.5% | 4.6% | 3.5% | 40.1% |
| **Married** | 4.7% | 8.1% | 29.4% | 11.2% | 6.5% | 59.9% |
| **Total** | 15.1% | 16.2% | 42.9% | 15.8% | 10.0% | 100.0% |
